# Influence of leadership empowering behavior on employee innovation behavior: The moderating effect of personal development support

**DOI:** 10.3389/fpsyg.2022.1022377

**Published:** 2022-12-19

**Authors:** Pinghao Ye, Liqiong Liu, Joseph Tan

**Affiliations:** ^1^School of Information Engineering, Wuhan Business University, Wuhan, China; ^2^DeGroote School of Business, McMaster University, Hamilton, ON, Canada

**Keywords:** leadership empowering behavior, participative decision making, thriving at work, employee innovation behavior, personal development support

## Abstract

The main purpose of this study is to explore the influence of leadership empowering behavior (personal development support, participative decision making and delegation of authority) and thriving at work (vigor, learning) on employee innovation behavior and analyze the moderating effect of personal development support on participative decision making and innovation behavior. The questionnaire survey method is used to survey Chinese industrial workers, and a total of 290 valid questionnaires are collected. The model is verified using SmartPLS. Results show that the personal development support and participative decision making dimensions of leadership empowering behavior have a significant positive influence on employee innovation behavior. Vigor and learning has a significant positive influence on employee innovation behavior, and personal development support has a significant moderating effect on the relationship between participative decision making and innovative behavior.

## Introduction

Independent innovation is the key for modern enterprises to build core competitiveness in a dynamic environment and related to their survival and development (Wang and Nickerson, 2017). Moreover, individual innovation is the foundation of organizational innovation (Zhao et al., 2021). As the main body of enterprise innovation, employees’ innovation behavior has a positive influence on enterprise innovation performance (Miron-Spektor and Beenen, 2015; Usai et al., 2020). Thus, research on employees’ innovation behavior has become a hot issue.

Early research on employees’ innovation behavior concentrated mostly on the field of psychology from the perspective of personal characteristics (Petrosyan, 2019), and subsequent research focused gradually on the influence of external situational factors (Zuraik et al., 2020). As an important part of the external context, leadership plays an important role in corporate innovation. Leadership style and manner can directly or indirectly influence employees to demonstrate innovative behavior. Through empowerment, leaders can make employees feel the company’s support and attention, which will lead to innovation behavior. Therefore, exploring how to improve employees’ innovative behavior from the perspective of leadership is essential. However, most studies started only from the perspective of leadership style, such as transformational, authentic, service-oriented and ethical leadership, to explore the influence of leadership on employees’ innovative behavior (Michaelis et al., 2010; Özsungur, 2019; Bagheri et al., 2020; Zhang et al., 2021b), and research on the effect of leadership empowerment on employees’ innovation behavior is limited. Although studies showed that leadership empowerment has a positive influence on employees’ innovation behavior, the action mechanism between the two factors is unclear. Does leadership empowerment directly or indirectly affect employees’ innovation behavior? What other factors exist in this influence? Previous studies failed to confirm such issues in detail.

In addition, numerous studies revealed that thriving at work plays an important role in the innovation behavior of employees, but limited research incorporated leadership empowering behavior and thriving at work into research models and explored their influence on innovation behavior at the same time. Moreover, does this role exist in the context of Chinese high-tech enterprises? Is it a positive or negative regulation? Employees’ innovation behavior is affected by not only the external leadership environment but also individual factors. Research at home and abroad showed that personal development support has a significant influence on employees’ innovation behavior (Rigopoulou and Kehagias, 2008), but previous research failed to provide a clear answer on how this influence works. In addition, participative decision making was confirmed by a large number of studies to have an influence on the innovation behavior of employees within an enterprise (Sarafidou and Chatziioannidis, 2013; Huang et al., 2015; Wilson, 2016; Salomé and Andrea, 2017), and delegation of authority typically emerges in the process of corporate management (Li L. et al., 2020; Liu X. et al., 2020). Enterprises maintain an open attitude towards power appointment, which will create the power appointment management atmosphere, and employees’ perception of a power appointment will have an influence on their innovation behavior.

Therefore, after defining the concepts of ‘leadership empowerment’ and ‘employee innovation behavior’, this study constructs a ‘leadership empowering behavior–thriving at work–employee innovation behavior’ research framework and introduces delegation of authority and participative decision making into the research framework. From the perspective of social cognition and empowerment theory, this study explores the influence path and mechanism of leadership empowerment on employees’ innovation behavior and determines whether personal development support can effectively regulate the relationship between participative decision making and employees’ innovation behavior. The conclusions of this study may have theoretical significance and guide management practice for research on employees’ innovation behavior to help leaders inspire employees’ innovation behavior and improve corporate innovation performance.

This paper is mainly divided into seven parts. The first section introduces the research background, significance and content of the influence of leaders’ empowering behavior on employees’ innovation behavior. The second section presents the literature review of research on employees’ behaviors empowered by leaders, thriving at work and innovation behavior. The third section constructs the research model of the influencing factors of leadership empowering behavior and thriving at work for employees’ innovation behavior and presents the research hypotheses. The fourth section systematically combs through the variables and measurement items included in the research model, describes the questionnaire and research methods and explains the data analysis techniques used in this research. The fifth section analyses the data of the collected valid questionnaires and draws the conclusions. The sixth section assesses and summarizes the research conclusions, and the seventh section identifies the research limitations and future research prospects.

## Background

### Leadership empowering behavior

Leadership empowering behavior was first proposed by Konczak et al. (2000) as a series of management behaviors adopted by leaders to empower employees. Leadership empowering behavior is a type of special leadership style differing from traditional leadership. Leadership empowering behavior occurs between a leader and a subordinate, emphasising the process of power sharing between a leader and employees and allowing employees to develop self-control and perform tasks autonomously (Vecchio et al., 2010; Sharma and Kirkman, 2015). In addition, leadership empowering behavior is an implementation process, the core of which involves a leader delegating power to employees, eliminating employees’ sense of powerlessness and enhancing employees’ autonomy to stimulate their intrinsic motivation and promote their development as well as that of the company (Thomas and Velthouse, 1990; Le and Wei, 2011). The essence of leadership empowering behavior is a series of management behaviors (Oedzes et al., 2019) to share information and rights with employees (Vecchio et al., 2010) and promote psychological empowerment to provide employees with increased opportunities to participate in decision making, which will improve their self-efficacy and work performance (Seibert et al., 2011; Auh et al., 2014).

Scholars conceptualized and verified the dimensions of this unique set of leadership behaviors and distinguished them from other related leadership structures. For example, Arnold et al. (2000) identified five key aspects of leadership empowering behavior, that is, leading by example, participative decision making, guiding, informing and mutual attention. Ahearne et al. (2005) analyzed four dimensions of leadership empowering behavior, specifically, delegation of authority, participative decision making, trust in subordinates and strengthening the meaning of work. Amundsen and Martinsen (2015) believed that the two core dimensions of leadership empowering behavior are independent support and development support. Meanwhile, Pearce and Sims (2002) argued that encouraging employees to develop their abilities, promoting employee autonomy, supporting employees to seek opportunities actively, attaching importance to teamwork, setting goals reasonably, and strengthening employees’ self-management should be the six important aspects of leadership empowering behavior. Konczak et al. (2000) identified six dimensions of leadership empowering behavior, namely, delegation of authority, support innovation, independent decision making, skills development, information sharing, and taking responsibility.

Although the aforementioned scholars adopted different perspectives on the dimensional division of leadership empowering behavior, numerous common points exist, which describe the empowering role of leadership empowering behavior and transform previous “management + control” behavior into “help guide”, “strengthening the meaning of work”, and “team interaction” to reshape the value of work. They transform from the abstract behavior of strengthening the “trust atmosphere” into the equal communication behavior of leaders’ “information sharing” and from previous “top-down” decentralization into leaders’ encouragement of employees to “participate in decision making”. Based on previous research, this study examines the influence of leadership empowering behavior on employees’ innovation behavior from the three dimensions of personal development support, participative decision making and delegation of authority and investigates the relationship between the three dimensions.

### Thriving at work

Thriving at work is a concept of active organizational behavior, including two dimensions, namely, vigor and learning. The vigor dimension examines whether employees feel energized and enthusiastic at work, and the learning dimension mainly examines whether employees have self-confidence from mastering knowledge or skills (Spreitzer et al., 2005; Wang et al., 2019). Compared with work investment, in addition to vigor, thriving at work places more emphasis on employee learning and growth experience. Research confirmed that a high degree of thriving at work energizes employees and gives them a sense of growth and a high level of innovation at work (Sia and Duari, 2018; Shahid et al., 2021). Therefore, enhancing employees’ sense of thriving at work is significant for improving their innovation behavior.

Existing empirical studies extensively verified the positive relationship between thriving at work and work performance (Cynthia et al., 2015; Frazier and Tupper, 2016; Walumbwa et al., 2017). Recently, a meta-analysis of 73 empirical research papers concluded that work exuberance has a predictive effect of 0.35 on work performance (Kleine et al., 2019). The positive mental state of thriving at work can generate increased positive experiences and resorces and improve work performance (Wang and Meng, 2021). According to the above research, empowering leadership is conducive to improving thriving at work, and a strong correlation exists between the two factors. Therefore, the present study includes thriving at work in the research scope. Learning and vigor are two key variables of thriving at work. Many scholars confirmed the positive influence of learning on employees’ innovation behavior (Víctor et al., 2008; Chung and Li, 2021). This study uses vigor and learning to represent employee prosperity and conducts research on employees’ innovation behavior.

### Innovation behavior

Kanter (1988) first pointed out that individual innovation behavior can be divided into three stages, from identifying problems and proposing solutions to forming groups to realize ideas and finally spreading the innovative results. This definition includes the initiation and result of innovation behavior rather than merely action. Scott and Bruce (1994) emphasized that employees’ innovation behavior involves the individual identification and understanding of problems and building an innovation team to put the innovative ideas into practice and finally commercializing the action of innovative practices. This process completes the generation, development and realization of ideas. Moreover, the process is a combination of a series of discontinuous activities, with different relatively independent innovation activities in each stage. Amabile et al. (1996) regarded innovation as a new idea, new scheme and work path that can bring value to an organization. Meanwhile, Kleysen and Street (2001) argued that innovation behavior should be understood comprehensively, from the initial discovery of opportunities to the initiation of ideas, multifaceted evaluation of innovation, creative support, and finally, the realization of the creative ideas. Shen et al. (2017) believed that the concept of employee innovation behavior is to generate innovative ideas at work and turn ideas into practice.

Kirton (1976) posited that employees’ innovation behavior is affected by their characteristics. If employees enjoy thinking about problems in accordance with their original path, then their innovation behavior will be minimal. However, if employees tend to find different ways to ponder problems, then they will demonstrate considerable innovative behavior. Innovation behavior involves not following the existing path, pondering a problem spontaneously and solving the problem in a unique way.

In summary, this study uses the viewpoint of Scott and Bruce (1994) to define employee innovation behavior as producing or adopting new methods, ideas and technologies and putting them into practice in the actual production activities of an organization to improve original management procedures or practices and enhance the organization’s production efficiency.

## Research model

Leadership exerts an important influence on employee innovation. An increasing number of studies showed that leadership is a key factor promoting innovation (Hammond et al., 2011; Miao et al., 2018), that is, support and encouragement from leaders have a significant positive influence on employees’ innovation. The more a leader delegates rights to employees, and the more the support and encouragement given to employees, the more the creativity demonstrated by the employees (Assen, 2020). Leadership empowering behavior emphasizes that employees share information and rights to gain opportunities to participate in decision making, strengthen their intrinsic motivation and stimulate their innovative behavior. In addition, leadership empowering behavior can enhance employees’ sense of belonging and commitment to the organization (Chung et al., 2011; Kundu et al., 2019) and improve their satisfaction at work, influencing them to think about the organization as much as possible, thereby improving their work performance (Chang, 2016; Idris et al., 2018; Gong et al., 2021) and generating increased innovation behavior. This outcome is conducive to an organization to generate other innovative activities (Kool and Dirk, 2012; Li et al., 2016). Therefore, this research examines the influence of leadership empowering behavior on employees’ innovation behavior from three aspects, that is, personal development support, participative decision making and delegation of authority, and proposes the following hypotheses:

*H1*: Personal development support has a significant positive influence on innovation behavior.

*H2*: Participative decision making has a significant positive influence on innovation behavior.

*H3*: Delegation of authority has a significant positive influence on innovation behavior.

The literature on employee innovation points out that participative decision making and personal development support are important prerequisites for generating innovative results (Amabile et al., 2004; Khan et al., 2021). Based on this idea, Amabile et al. (1996) found that leaders’ empowering behavior can give employees increased decision-making power and opportunities to make choices by delegating rights to employees and enabling them to make decisions and take action without direct supervision or intervention. This approach can encourage and empower employees to explore various creative solutions before determining feasible solutions and improve the output efficiency of innovation results. Zhang and Bartol (2010) conducted an empirical analysis to verify the influence of leadership empowering behavior on employees’ innovation behavior and determined that leadership empowering behavior can increase employees’ enthusiasm to solve problems, give them considerable freedom, stimulate their creativity and promote their innovation.

At the same time, social exchange theory asserts that leaders can establish high-quality reciprocal exchange relationships with employees through delegation of authority, personal development support, encouragement to participate in decision making and work guidance, which can promote employees’ positive behavior and generate positive results for the organization (Erkutlu and Chafra, 2015). Therefore, based on the literature on leadership empowering behavior and social exchange theory, this study divides leadership empowering behavior into three dimensions, that is, personal development support, participative decision making and delegation of authority, and proposes the following hypotheses:

*H4*: Participative decision making has a significant positive influence on personal development support.

*H5*: Participative decision making has a significant positive influence on delegation of authority.

Thriving at work is a psychological state of an employee and obtained through learning and by experiencing vigor at work. Learning refers to the enhancement of self-confidence and strength through knowledge and skills, and vigor represents employees’ high level of energy at work (Amabile, 1988; Spreitzer et al., 2005; Endrejat, 2021). Empowering leadership can help employees build confidence, encourage them to try new methods and promote continuous learning (Fry et al., 2005; Hirak et al., 2012; Meng, 2016). In addition, empowering leadership invites employees to participate in corporate management decisions and allows them to express different opinions, thereby giving them sufficient rights to solve problems and increasing their enthusiasm for work. Furthermore, empowering leadership pays attention to employees’ sense of happiness at work and enhances their sense of belonging by satisfying their communication and emotional needs, thereby improving their work vigor (Sorakraikitikul and Siengthai, 2014; Lei et al., 2021). Employees’ self-confidence, work autonomy and sense of belonging are conducive to stimulate their sense of thriving at work.

Employees’ innovative and proactive behaviors have obvious characteristics based typically on a positive and optimistic work attitude, and thriving at work reflects employees’ positive emotional state. When employees experience positive emotions generated through learning and vigor at work, they will deeply ponder their activities and promote the generation of innovative ideas (Jiang et al., 2020). Employees promote the construction of their resource system and innovation behavior through learning and by maintaining vigor at work (Isgett and Fredrickson, 2004; Saxena et al., 2020). Therefore, when employees have a high sense of thriving at work, they will have a strong desire to gain new knowledge and skills and will be able to use new methods and technologies in various ways to engage in challenging work and adapt to the dynamic needs of their organization (Guan and Frenkel, 2020). Employees with a high sense of thriving at work tend to spread new knowledge and skills in the organization and expend a considerable amount of energy on practice, which can promote their innovation performance. When employees have a sense of thriving at work, they can improve their innovation ability through active learning and by maintaining their vigor and demonstrate innovative proactive behavior exceeding the requirements of their work (Lee and Lee, 2020). Therefore, this study proposes the following hypotheses:

*H6*: Delegation of authority has a significant positive influence on vigor.

*H7*: Vigor has a significant positive influence on innovation behavior.

*H8*: Learning has a significant positive influence on innovation behavior.

The effect of empowering leadership on employees’ personal development support will enhance employees’ intrinsic motivation and willingness to engage in complex, creative, proactive and self-directed activities (Frascaroli et al., 2015). In addition, it can enhance sense of effectiveness of employees’ role width perception, increase their confidence in performing comprehensive tasks outside of work and improve their proactive work performance (McDonald et al., 2012; Zhang et al., 2021a). In the process of participating in decision making, employees will sense their leaders’ support for their personal development (Band et al., 2019). Based on this concept, this study proposes the following hypothesis:

*H9*: Personal development support has a significant moderating effect on the relationship between participative decision making and innovative behavior.

## Research methodology

### Sampling and data collection

This study uses a questionnaire survey to conduct empirical research and collect data, with industrial workers in China as the research object. The employees selected for this study refer mainly to technical employees, junior managers, middle managers, senior managers and employees engaged mainly in product design, research and development and testing. Such employees are the main members of enterprise innovation and play an important role in an enterprise. According to unified standards and requirements, the questionnaire is mainly distributed online through the WenJuanXing (WJX) data collection platform. WJX is an online research platform based in Changsha, China. With questionnaires, the platform collects data for economic management, psychology and education; provides powerful data storage and analysis functions; and digs deeply into the value of the data. Provide convenient data collection tools for scientific researchers.

In this survey, a total of 415 questionnaires are distributed, and 290 valid questionnaires are obtained, with an effective rate of 70%. The descriptive statistics show that in the effective sample, the male respondents account for 45.9%, and the female respondents account for 54.1%. For the age distribution, the respondents 25 years old and below account for 33.4%, those between the ages of 26 and 35 years account for 45.2%, those between the ages of 36 and 45 years account for 16.2% and those 46 years old and above account for 5.2%. The unmarried respondents account for 60.3%, and the married respondents account for 39.7%. Those employed for less than 2 years account for 24.8%, and those employed for 2 to 5 years account for 26.6%. The respondents employed for 6 to 9 years account for 23.4%, and those employed for over 10 years account for 25.2%. For the education distribution, the respondents who reached junior college and below account for 19%, those with a bachelor’s degree account for 70%, those with a master’s degree account for 7.9% and those with a doctoral degree and above account for 3.1%. For job distribution, technical staff account for 32.1%, junior management staff account for 35.5%, middle management staff account for 27.6% and senior management staff account for 4.8%. The respondents with an income below RMB 4,000 account for 21%, those with an income of RMB 4,001–5,000 account for 16.2%, those with an income of RMB 5,001–6,000 account for 11%, those with an income of RMB 6,001–7,000 account for 9.7%, those with an income of RMB 7,001–8,000 account for 14.5% and those with an income of RMB 8,000 or more account for 27.6%. The basic information of the survey object is shown in Table 1.

**Table 1:** Demographic characteristics of valid sample.

Variables	Categories	Frequency	Percentage
Gender	Male	133	45.9%
	Female	157	54.1%
Age	25 years and below	97	33.4%
	26–35 years	131	45.2%
	36–45 years	47	16.2%
	46 years and above	15	5.2%
Marital status	Unmarried	175	60.3%
	Married	115	39.7%
Years employed	2 years and below	72	24.8%
	2–5 years	77	26.6%
	6–9 years	68	23.4%
	10 years or more	73	25.2%
Education level	Junior college and below	55	19.0%
	Bachelor’s degree	203	70.0%
	Master’s degree	23	7.9%
	Doctoral degree	9	3.1%
Position	Technician	93	32.1%
	Junior management	103	35.5%
	Middle management	80	27.6%
	Senior management	14	4.8%
Title	Junior	134	46.2%
	Middle	125	43.1%
	Subsenior	23	7.9%
	Senior	8	2.8%
Monthly income	RMB 4,000 and below	61	21.0%
	RMB 4,001–5,000	47	16.2%
	RMB 5,001–6,000	32	11.0%
	RMB 6,001–7,000	28	9.7%
	RMB 7,001–8,000	42	14.5%
	RMB 8,000 and above	80	27.6%

### Questionnaire and measurements

The measurement questionnaire is based mainly on mature scales, On the basis of the research results of scholars, 18 influencing factors of employee innovation behavior were extracted from the literature, and the initial measurement scale was formed. and the research design is carried out in strict accordance with the translation-back translation procedure. On this basis, appropriate adjustments are made according to the Chinese context. In order to verify and supplement the existing research, representative enterprises were selected for in-depth interviews. Firstly, We state the understanding of relevant scholars on the connotation, extension and influencing factors of employee innovation behavior, and ask them to explain whether the existing research results can be established in the enterprise based on the actual situation of the enterprise. Through interviews, the factors extracted from the literature were confirmed in the enterprise.

Based on this interview, a presurvey is conducted, and the questionnaire is revised and improved based on the presurvey feedback to create the formal questionnaire, except for the basic situation of the staff. In addition, the influence of leadership empowering behavior and thriving at work on employees’ innovation behavior is investigated. The questionnaire uses a seven-point Likert scale, with 1 representing “completely disagree” and 7 representing “completely agree”.

The questionnaire is revised based on the leadership empowering behavior scale compiled by Arnold et al. (2000), Konczak et al. (2000), Slåtten et al. (2011), Hassi (2019), Naqshbandi et al. (2019) combining the characteristics of employees’ innovation behavior. Based on the feedback and presurvey results, three items, that is, *Personal Development Support*, *Participative Decision Making* and *Delegation of Authority*, are determined (Mutonyi et al., 2020). This study draws on the scale of Shirom (2003), Porath et al. (2012), Duan et al. (2021) to create the vigor and learning. Moreover, this study draws on the scale of Porath et al. (2012), Spreitzer et al. (2012), Spanuth and Wald (2017) to measure employees’ innovation behavior. The last measurement items of the five constructs are listed in the Appendix.

### Extraction of main factors

Numerical KMO calculation and Bartlett spherical test were performed for sample data. The KMO value of the scale was 0.909, indicating that the sample adequacy was high and suitable for progressive factor analysis. The $\;x^{2}$ value of Bartlett spherical test was 1637.339 (153 degrees of freedom), and the accompanying probability was 0.000, less than 0.05, indicating that there was correlation between the items of the scale, which was suitable for factor analysis.

The principal component analysis method was used to extract the main factors, and the factors with eigenvalue greater than 1 were selected. The maximum variance method was used to rotate the factors, and the items that were self-contained as one factor and the load values of two or more factors were all less than 0.5 were deleted. Two factor analyses were conducted. A total of 5 items were deleted, namely QPDS3, QPDS5, QTW3, QTW4 and QTW5. After the items were deleted, the KMO value of the scale was 0.896, the $\;x^{2}\;$ value of Bartlett spherical test was 1209.206 (with 78 degrees of freedom), and the accompanying probability was 0.000, less than 0.05. It shows that there are common factors among the correlation matrices of the mother, indicating that the data are suitable for factor analysis. A total of 4 factors are extracted, and the cumulative variance contribution rate is 51.975%, which can explain most of the structure of the original variable and reflect most of the information of the original variable. Thus, four main factors of influencing factors of employee innovation behavior are obtained. Exploratory factor analysis was completed.

### Reliability and validity tests

Cronbach’s α reliability coefficient was used to test the internal consistency of the scale. After deleting 5 items, the Cronbach’s α coefficient of the scale as a whole was 0.870, indicating that its reliability and stability were good and its reliability was high. The Cronbach ‘α of the four subscales is greater than 0.6, indicating that they also have good reliability.

This study uses SPSS 24.0 to test the reliability and validity of *Personal Development Support*, *Participative Decision Making*, *Delegation of Authority*, *Vigor, Learning* and *Innovation Behavior*, and the results are presented in Table 2. It can be seen from Table 2 that the composite reliability (CR) of each latent variable is greater than 0.75, and the Cronbach’s α coefficient values are all greater than the recognized lowest level of 0.6, thereby indicating that the scales demonstrate satisfactory reliability. Exploratory factor analysis is used to test the structural validity of the scales, and the factor loading of each item corresponding to all the variables is greater than the threshold of 0.7, thereby indicating that the scales have satisfactory structural validity (Nunnally, 1978).

**Table 2:** Reliability analysis.

Construct	Items	Factor Loading^a^	Cronbach’s α	CR	Average Variance Extracted (AVE)
Personal development support (PDS)	PDS1	0.761	0.738	0.836	0.560
	PDS2	0.771			
	PDS3	0.738			
	PDS4	0.723			
Participative decision making (PDM)	PDM1	0.783	0.763	0.849	0.584
	PDM2	0.759			
	PDM3	0.779			
	PDM4	0.735			
Delegation of authority (DOA)	DOA1	0.744	0.715	0.839	0.636
	DOA2	0.874			
	DOA3	0.769			
Vigour (VI)	VI1	0.883	0.715	0.875	0.778
	VI2	0.881			
Learning (LE)	LE1	0.718	0.551	0.769	0.526
	LE2	0.700			
	LE3	0.758			
Innovation behavior (IB)	IB1	0.754	0.616	0.795	0.565
	IB2	0.739			
	IB3	0.761			

The AVE of each variable is greater than 0.5, thereby indicating that the scales have satisfactory convergent validity (Fornell and Larcker, 1981). Combining Table 3, the square root of the AVE of each variable is greater than the correlation coefficient between the variable and the other variables. The variables exhibit satisfactory discriminant validity, which shows that the scales used in this paper demonstrate satisfactory validity (Hair et al., 1998).

**Table 3:** Validity analysis.

	Personal development support	Innovation behavior	Participative decision making	Learning	Delegation of authority	Vigour
Personal development support	0.748					
Innovation behavior	0.483	0.751				
Participative decision making	0.627	0.550	0.764			
Learning	0.458	0.500	0.390	0.726		
Delegation of authority	0.469	0.325	0.593	0.255	0.798	
Vigour	0.504	0.476	0.446	0.379	0.324	0.882

## Data analysis and results

Partial least squares (PLS) are used to analyze the model. This technique is a new type of multivariate data analysis method, with more reliable and stable calculation results compared with other methods. In addition, this method is suitable for analyzing small data samples and can simultaneously realize modelling prediction, the comprehensive simplification of multivariable systems and correlation analysis between two sets of variables, which can effectively solve the problem of collinearity. The main purpose of this method is to build a regression model between multiple dependent and independent variables (Chin et al., 2020). Moreover, when constructing the model, PLS can set the external relationship type in the structural equation flexibly according to the actual situation, that is, it supports the constitutive model and reflective model (Richter et al., 2020). SmartPLS 3.0 is used in this study to analyze the model.

### Path coefficient and hypothesis test

**Figure 1:**
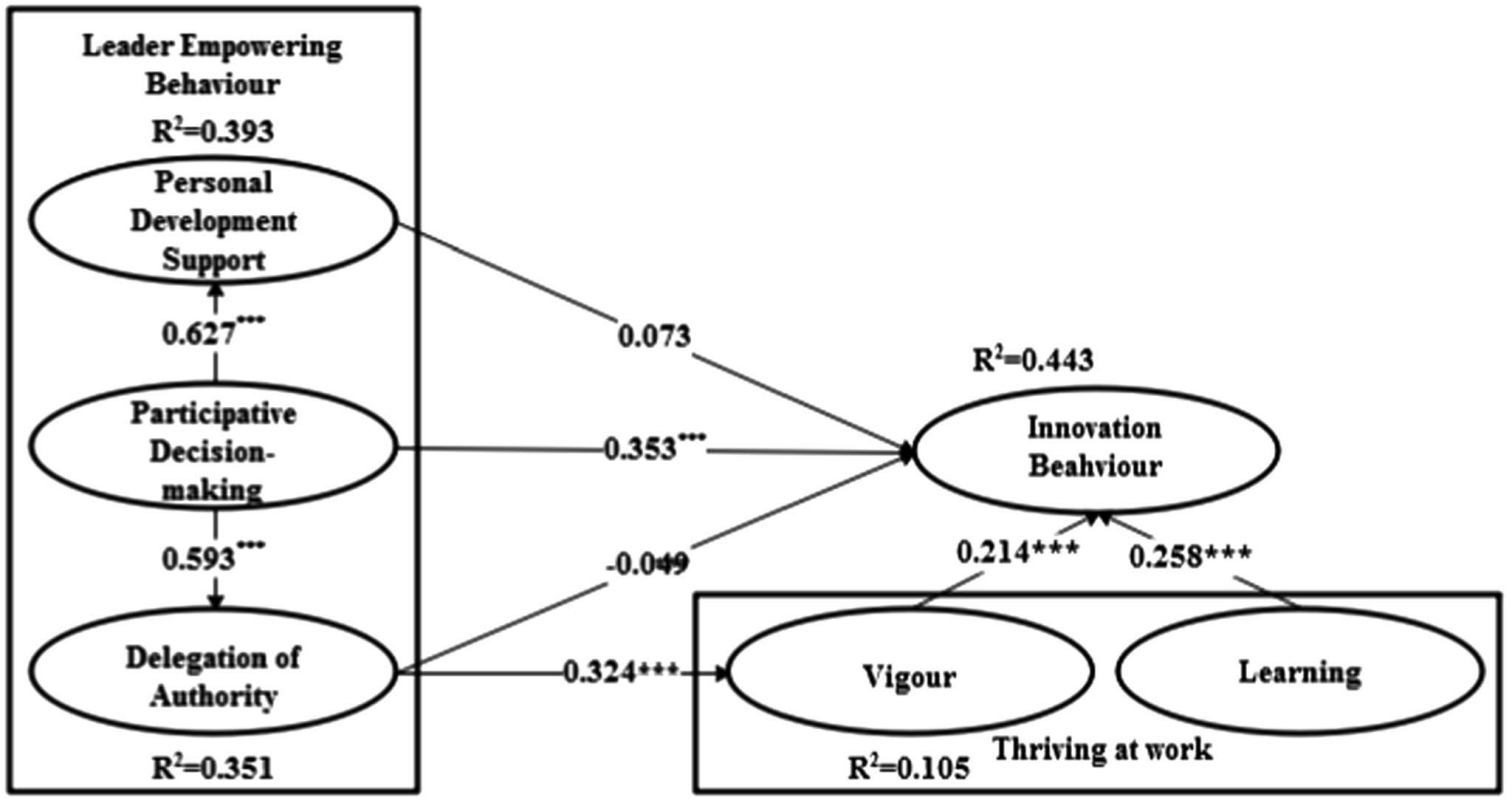
Model path and significance level.

The path coefficient indicates the strength of the relationship between the independent and dependent variables (Thom, 1983). The results of the path coefficient analysis of the study model are presented in Figure 1 and Table 4. All seven hypotheses are supported.

**Table 4:** Hypothesis testing results.

Hypothesis	Path	Mean	SD	*t*-value	*p*	Supported
H1	PDS → IB	0.071	0.068	1.069	0.285	No
H2	PDM → IB	0.355	0.082	4.279	0.000	Yes
H3	DOA → IB	−0.046	0.064	0.762	0.446	No
H4	PDM → PDS	0.630	0.039	15.897	0.000	Yes
H5	PDM → DOA	0.595	0.040	14.647	0.000	Yes
H6	DOA → VI	0.328	0.057	5.674	0.000	Yes
H7	VI → IB	0.212	0.057	3.750	0.000	Yes
H8	LE → IB	0.263	0.056	4.606	0.000	Yes

R^2^ is the variance variability explained by the dependent variable. In this study, the bootstrapping repeated sampling method is used to select 3,000 samples to calculate the *t*-value of the significance test. The interpretation degree of *Personal Development Support*, *Delegation of Authority* and *Innovation Behavior* is 0.393, 0.351 and 0.395, respectively, thereby indicating that the model has a satisfactory interpretation effect.

In this study, the bootstrapping method is used to test the significance of the path coefficients of the structural model, and the results are shown in Table 4. The effect of *Personal Development Support* on *Innovation Behavior* is unverified (*β* = 0.073, *t* = 1.069), thus, H1 is unconfirmed. *Participative Decision Making* has a significant positive influence on *Innovation Behavior* (*β* = 0.396, *t* = 4.925), thereby supporting H2. The effect of *Delegation of Authority* on *Innovation Behavior* is unverified (*β* = −0.051, *t* = 0.852); thus, H3 is unconfirmed. *Participative Decision Making* has a significant positive influence on *Personal Development Support* (*β* = 0.630, *t* = 16.204), thereby supporting H4. *Participative Decision Making* has a significant positive influence on *Delegation of Authority* (*β* = 0.595, *t* = 14.421), thereby supporting H5, and *Delegation of Authority* has a significant positive effect on *Vigor* (*β* = 0.326, *t* = 5.595), thereby supporting H6. *Vigor* has a significant positive effect on *Innovation Behavior* (*β* = 0.326, *t* = 4.714), thereby supporting H7. *Learning* has a significant positive effect on *Innovation Behavior* (*β* = 0.258, *t* = 4.606), thereby supporting H8.

### Moderating effect test

To test the moderating effect of *Personal Development Support* on the relationship between *Participative Decision Making* and *Innovation Behavior*, hierarchical regression analysis is employed. This study investigated the role of variables at the level of Personal development support and Participative decision-making on the dependent variable. On this basis, it continues to investigate whether the variable Participative decision-making will affect the slope between the independent variable and the dependent variable at the Personal development support level, so as to obtain the slope prediction model, namely the full model.

**Figure 2:**
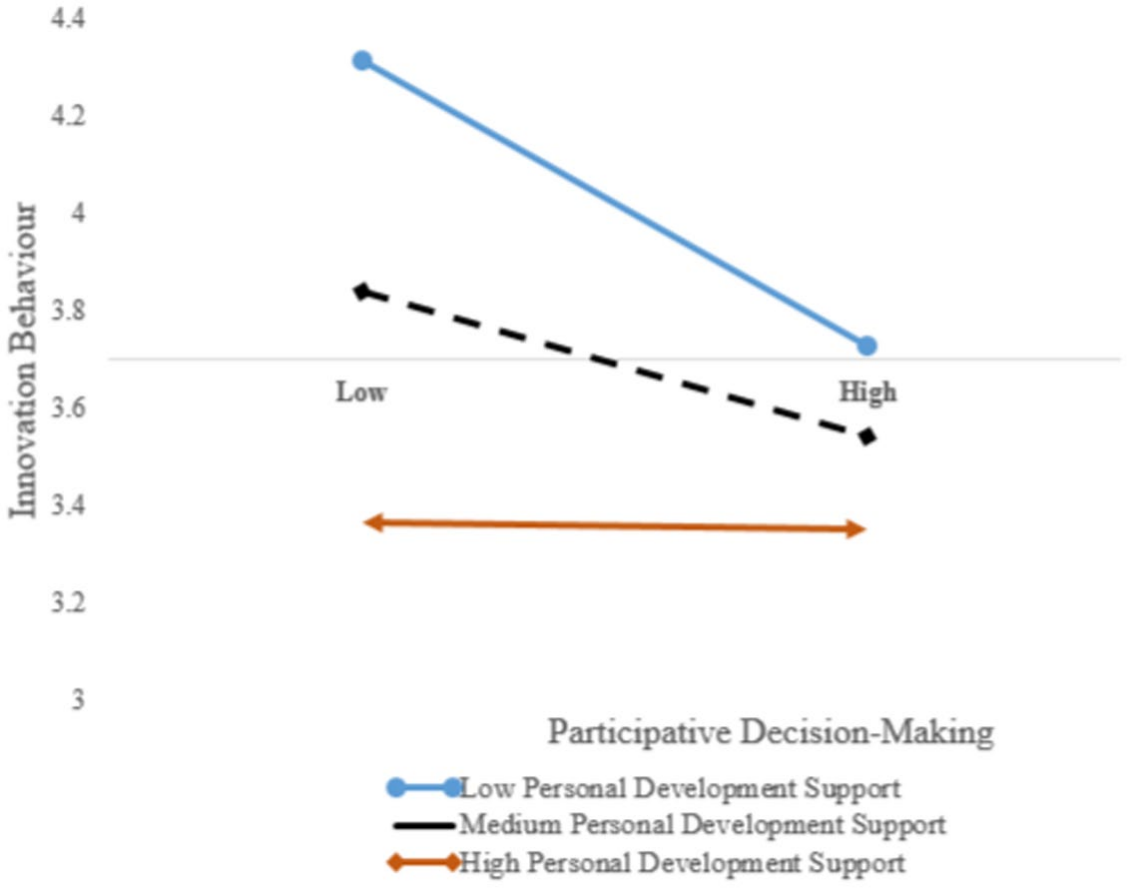
Moderating effect of *Personal Development Support* on the relationship between *Participative Decision Making* and *Innovation Behavior.*

Before verifying the moderating effect, centralising the variables of the cross terms to avoid collinearity is necessary. Next, the variables processed through centralization are multiplied to construct the interactive items. In this study, the independent and adjusted variables are processed centrally to construct the product terms of *Personal Development Support* and *Participative Decision Making* with *Innovation Behavior* for the multilevel regression analysis. *Personal Development Support* has a significant regulatory effect on the relationship between *Participative Decision Making* and *Innovation Behavior*. In the study, *Personal Development Support* is divided into high, medium and low conditions, which can facilitate the clear display of the role of the regulatory variables. Excel is used to plot the degree of influence of *Participative Decision Making* on *Innovation Behavior* in the high, medium and low conditions of *Personal Development Support*. The main effect (*Participative Decision Making*) is −0.15, the moderating variable effect (*Personal Development Support*) is −0.331 and the moderating effect (*Participative Decision Making* × *Personal Development Support*) is 0.143, *p* = 0.023. The moderating effect is shown in Figure 2, and the results reveal that when *Personal Development Support* is high, the influence of *Participative Decision Making* on *Innovation Behavior* increases, thereby supporting H8. Personal development support has a significant moderating effect on the relationship between participative decision making and innovative behavior.

## Discussion and conclusion

### Discussion of findings

This study analyses the factors influencing employee innovation behavior from two aspects, namely, leadership empowering behavior and thriving at work. The following key conclusions are drawn.

Firstly, the personal development support and participative decision making dimensions of leadership empowering behavior have a significant positive influence on employees’ innovation behavior, but the influence of delegation of authority on employees’ innovation behavior is unconfirmed. The conclusions of this study indicate that companies should gradually shift their leadership style from centralization to authorization. Leadership empowerment can help employees share increased resources (Soliman, 2020); make employees feel the support, attention and encouragement of the company; and enhance their sense of belonging and loyalty, thereby improving their sense of innovation self-efficacy and further stimulating their innovation behavior (Cheong et al., 2019). Therefore, under a stable corporate organizational structure, leadership empowerment is conducive to corporate development.

Secondly, thriving at work has a significant positive influence on innovation behavior. This outcome shows that vigor and learning can influence employees’ creativity and help them form, maintain and develop their creativity. According to componential theory of creativity, ability, knowledge and motivation are the key internal components of creativity (Rukhsar, 2015). Employees with a high sense of thriving at work have a high level of knowledge, vigor and energy. Through continuous learning and by honing and improving their professional abilities and skills, employees can generate innovative ideas (Gundry et al., 2016).

Thirdly, the participative decision making dimension of leadership empowering behavior has a significant positive influence on personal development support and delegation of authority. At the same time, delegation of authority has a significant positive influence on vigor. The research conclusions show that the participative decision making dimension of leadership empowering behavior can satisfy employees’ sense of participation. By participating in the company’s decision making, employees’ dominant position is respected, and increased psychological capital is obtained (Erkutlu and Chafra, 2015; Wu and Chen, 2015). In the process of participating in decision making, employees can easily obtain support from their leaders for their personal development by providing reasonable suggestions and innovative ideas (Wu and Chen, 2015). When employees are adequately capable, leaders will consider granting rights and appointments to facilitate increased innovative work.

Finally, personal development support has a significant moderating effect on the relationship between participative decision making and innovative behavior.

The analysis finds that the relationship between employees’ participative decision making and innovation behavior is affected by personal development support. When leaders’ support for employees’ personal development is high, it can stimulate employees’ enthusiasm for work (Huo and Jiang, 2021), thereby encouraging them to participate actively in the development of the enterprise, express practical innovation views and provide innovation experiences and innovation models for the innovation and development of the enterprise (Kremer et al., 2019) and enhancing the overall innovation atmosphere of the enterprise and employees’ innovation behavior.

### Practical implications

The important insights of this research into the practice of business management mainly include the following aspects.

Firstly, leadership is one of the most important factors influencing employees’ innovation behavior. This study confirms the positive effect of leadership empowering behavior on employees’ innovation behavior and provides certain insights into how leaders can improve subordinates’ innovation through their own actions (Tian et al., 2015). In an enterprise, leaders must first determine the quality of the employees, confirm that their quality can match the power granted them and avoid abuse and waste of power that employees are unable to master. To stimulate employees’ innovation behavior, leaders must master the art of empowerment and delegate authority appropriately to enable employees to complete their work independently, understand the importance of responsibility and gain power whilst performing their corresponding obligations. Leaders must also regard employees’ development as the organization’s development and work as hard as possible to realize their value.

Secondly, leaders should focus on helping employees grow when they stimulate employees’ innovation behavior through their empowering behavior, such as helping them plan and ensure their career path. Leaders should constantly pay attention to employees’ work progress to prevent them from losing self-control after gaining decision-making power, which may cause delays, deviations and other consequences. An organization’s strategy and vision are decomposed into strategies at every level and conveyed correctly to subordinates to prevent them from deviating from the general direction of the organization and acting contrary to the organization’s expectations, thereby allowing them to participate in the organization’s decision-making process, especially in decisions related closely to themselves, such as the establishment of work goals for the following quarter, work standards and so on. Finally, leaders should communicate and maintain close contact with employees at all times to ensure the normal flow of information. This correct exercise of empowering behavior can expand employees’ resources and enhance their work, overall planning and leadership abilities. When employees are grateful and give back to the organization, they demonstrate increased innovation behavior, which will benefit the organization.

Thirdly, thriving at work has a substantial influence on employees’ innovation behavior. In management practice, leaders must pay attention to employees’ learning and growth and promote their learning and vigor. With the rapid development of science and technology, whether enterprises can advance is the key to their survival. The development of an enterprise is based on the development of its employees; thus, employees’ individual learning is directly related to the competitive advantage of the organization. This research confirms the correlation between thriving at work and innovation behavior. Employees can trigger their increased innovation behavior by mastering new knowledge and technology or continuously gaining and integrating existing knowledge and technology. Therefore, leaders should adopt other leadership methods that encourage employees to participate in decision making, guide and help employees and share information to stimulate employees’ vigor and learning behavior. At the same time, leaders and human resource departments should pay attention to guiding and promoting employees’ learning behavior in a variety of ways, such as training in the latest knowledge of the industry, knowledge sharing meetings within the organization, regular basic knowledge and skills examinations, inviting internal and external lecturers to teach employees and so on to establish a learning organization, create a positive environment for employees’ learning behavior, increase the availability of learning resources and help employees produce increased innovation behavior at work.

### Theoretical implications

Through the discussion, research and verification of the relationship between leadership empowering behavior, thriving at work and employees’ innovation behavior, this study makes the following theoretical contributions.

Firstly, most studies on leadership empowering behavior explored employees’ perception and proved that leadership empowering behavior can promote employees’ positive behavior (Javed et al., 2018). However, this research angle is broad, and the focus is narrow. This study is based on existing research results on personal development support, participative decision making and delegation of authority and analyses the influence of leadership empowering behavior on employees’ innovation behavior. Moreover, this study further explores the internal mechanism between the three dimensions, which enriches research in the field of not only leadership empowering behavior but also innovation to a certain extent.

Secondly, thriving at work is a positive human and social capital. This study introduces thriving at work into research on the relationship between leadership empowering behavior and employees’ innovation behavior, thereby verifying the influence of thriving at work on employees’ innovation behavior and proving the positive influence of delegation of authority on thriving at work (Basharat et al., 2018). In addition, this study enriches the theoretical basis of previous research on employees’ sense of thriving at work.

Thirdly, personal development support has a positive effect on employees’ innovation behavior and a significant moderating effect on the relationship between participative decision making and innovation behavior. This study introduces personal development support and examines its moderating effect, expands the boundary conditions of leadership empowering behavior and deepens understanding of the relationship between leadership empowering behavior and employees’ innovation behavior. Furthermore, this study enriches the theoretical basis of the mechanism of the leadership behavior style affecting employees’ innovation behavior.

## Limitations and future research directions

Although this research achieved certain results, deficiencies remain in some aspects. Firstly, the sample is limited. The majority of the sample is from China, and the depth and breadth of the sample are inadequate. In future studies, researchers should expand the research scope to other regions, increase the number of research enterprises, enrich the industry type, reduce the sample measurement errors and improve the reliability of the research conclusions. Secondly, this study explores only the influence of leadership empowering behavior on employees’ innovation behavior at the individual level. However, employees’ innovation behavior is also closely related to the organizational level and team level. In future research, increased consideration should be given to the influence of factors at other levels. Thirdly, the measurement scales used in this research are mature, but some adjustments were made in the specific application process, and the understanding of some issues is shallow, which may have a certain influence on the effectiveness of the research results. From the perspective of research method, The disadvantage of cross-sectional design is the lack of systematic and continuity. Because in cross-sectional design, each person is only examined at a certain point in time, it is impossible to obtain the data of individual development trend or development change. There is no continuity in the development of the same individual; Age and birth date cannot be distinguished; The sampling is complicated. Cross-sectional design at the same time has a cohort effect on subjects of different ages (Spector, 2019). In future studies, increased attention should be paid to adjusting and revising the scales based on specific cultural backgrounds to obtain accurate results and enhance the validity and persuasiveness of the conclusions.

## Data availability statement

The datasets presented in this study can be found in online repositories. The names of the repository/repositories and accession number (s) can be found in the article/ Supplementary material.

## Ethics statement

Ethical review and approval was not required for the study on human participants in accordance with the local legislation and institutional requirements. Written informed consent from the participants was not required to participate in this study in accordance with the national legislation and the institutional requirements.

## Author contributions

PY: data curation, formal analysis, methodology, writing—original draft, and writing—review and editing. LL: data curation, methodology, and writing—original draft. JT: data curation, writing—review and editing. All authors contributed to the article and approved the submitted version.

## Funding

This work was supported by the Hubei province technical innovation project (Soft Science Research) [2019ADD160].

## Conflict of interest

The authors declare that the research was conducted in the absence of any commercial or financial relationships that could be construed as a potential conflict of interest.

## Publisher’s note

All claims expressed in this article are solely those of the authors and do not necessarily represent those of their affiliated organizations, or those of the publisher, the editors and the reviewers. Any product that may be evaluated in this article, or claim that may be made by its manufacturer, is not guaranteed or endorsed by the publisher.

## Appendix

### Personal development support

1. My supervisor is very concerned about my personal growth and career plans.
2. My supervisor often provides me with training and learning opportunities.
3. My supervisor will try to get me promoted because of my outstanding job performance.
4. My supervisor often creates opportunities for me to show up and exercise.

### Participative decision making

1. When encountering problems at work, my supervisor actively listens to my opinions and suggestions.
2. When making decisions, my supervisor respects and values my suggestions.
3. My supervisor often creates opportunities for me to fully express my opinions.
4. In terms of my work and personal situation, my supervisor will ask for my opinion before making a decision.

### Delegation of authority

1. My supervisor does not interfere with the work within my scope of authority.
2. My supervisor is fully authorised to let me take full responsibility for the work I undertake.
3. My supervisor authorises me to make independent work decisions.

### Vigor

1. I am full of vigour at work.
2. I am often energetic at work.

### Learning

1. As time goes on, I learn more and more knowledge.
2. I think I’m constantly improving at work.
3. I can get more development in my job.

### Innovation behavior

1. I will facilitate the exchange of innovative ideas within the organization.
2. I will guide important people in the organization to become interested in innovative ideas.
3. I will turn innovative ideas into actual practices.
